# Machine Learning-Assisted SHG Morphometry Reveals Distinct Collagen Microarchitectures of Trabecular Bone and Fibrosis in Bone Marrow Biopsies

**DOI:** 10.3390/ijms27177685

**Published:** 2026-08-27

**Authors:** Zakhar P. Asaulenko, Anton A. Egorchev, Dmitry A. Peshekhonov, Leniz F. Nurullin, Anastasiia A. Melnikova, Alexander A. Rosin, Daria S. Vedischeva, Samat M. Shaidullin, Ilsaf I. Vafin, Anton S. Buchaka, Nikita S. Gladyshev, Maxim E. Fedchenko, Dmitry E. Chickrin, Yuriy A. Krivolapov, Dmitry V. Samigullin, Albert V. Aganov, Mikhail Paveliev

**Affiliations:** 1 Institute of Computational Mathematics and Information Technologies, Kazan Federal University, Kremlyovskaya 35, 420008 Kazan, Russia; zakhariy@list.ru (Z.P.A.); anton@egorchev.ru (A.A.E.); leniz2001@mail.ru (L.F.N.); anastasamelnikova@kpfu.ru (A.A.M.); alearosin@kpfu.ru (A.A.R.); ilsafivafin@kpfu.ru (I.I.V.); dmitry.kfu@ya.ru (D.E.C.); samid75@mail.ru (D.V.S.); 2 Pathological Anatomy Department, Federal Scientific and Clinical Center of Infectious Diseases of the Federal Medical and Biological Agency, 197022 St. Petersburg, Russia; 3 Pathological Anatomy Department of Clinical Molecular Morphology, E.E. Eichwald Clinic, North-Western State Medical University Named After I.I. Mechnikov, 195067 St. Petersburg, Russia; fedchenkomax93@mail.ru (M.E.F.); krivolapov.yuri@gmail.com (Y.A.K.); 4 Department of Medical Biology and Genetics, Kazan State Medical University, Butlerova 49, 420012 Kazan, Russia; peshehonovda@gmail.com (D.A.P.); nuka488@mail.ru (S.M.S.); 5 Kazan Institute of Biochemistry and Biophysics, FRC Kazan Scientific Center, Russian Academy of Sciences, Lobachevskogo 2/31, 420111 Kazan, Russia; 6 Institute of Physics, Kazan Federal University, Kremlyovskaya 16a, 420008 Kazan, Russia; alvaraganov@gmail.com; 7 Institute of Fundamental Medicine and Biology, Kazan Federal University, Karl Marx 74, 420015 Kazan, Russia; dvedom24@gmail.com; 8 Russian Scientific Center of Surgery Named After Academician B.V. Petrovsky, 2 Abrikosovsky Lane, 119991 Moscow, Russia; abpao62@yandex.ru (A.S.B.); krinege@mail.ru (N.S.G.); 9 Institute of Artificial Intelligence, Robotics and Systems Engineering, Kazan Federal University, Kremlyovskaya 18, 420008 Kazan, Russia; 10 Department of Sports Engineering, Kazan National Research Technical University Named After A.N. Tupolev-KAI, 10 K. Marx St., 420111 Kazan, Russia

**Keywords:** second harmonic generation microscopy, bone marrow fibrosis, trabecular bone, collagen microarchitecture, shallow machine learning, LabKit segmentation, histopathology, myelofibrosis, extracellular matrix remodeling, skeletonization

## Abstract

Collagen microarchitecture in bone marrow biopsies represents a largely underexplored source of candidate quantitative biomarkers for histopathological diagnostics and analysis of tissue remodeling. While second harmonic generation (SHG) microscopy has been increasingly applied to fibrosis assessment, the collagen organization of trabecular bone in bone marrow trephine biopsies remains poorly characterized. Here, we combined high-resolution SHG microscopy with shallow machine learning-assisted morphometry to compare collagen architecture in structured trabecular bone, unstructured trabecular bone, and fibrosis in bone marrow biopsies from patients with primary myelofibrosis. SHG image segmentation was performed using the LabKit plugin in Fiji. Several annotation strategies were evaluated to identify classifier configurations that preserved fibrillar structures. Quantitative morphometric analysis revealed marked differences in collagen organization between tissue types. Per-patient analysis consistently demonstrated thinner collagen fibers and reduced branching complexity in fibrosis than in structured trabecular bone. In contrast, unstructured trabecular bone showed extensive network branching accompanied by shorter skeleton branch length, consistent with remodeling-associated alterations of trabecular collagen architecture. Our results further demonstrate that annotation strategy substantially influences segmentation outcome and downstream morphometric measurements in SHG-based collagen analysis. Overall, this descriptive proof-of-concept study establishes a reproducible workflow for machine learning-assisted SHG morphometry that may prove useful for quantitative assessment of fibrosis, bone remodeling, and extracellular matrix organization in bone marrow pathology.

## 1. Introduction

Second harmonic generation (SHG) microscopy has emerged as a powerful label-free imaging modality for the quantitative characterization of collagen organization in biological tissues [1,2,3]. Biomedical applications of SHG collagen imaging span major histopathological domains including skin [4,5], liver fibrosis and steatohepatitis [6,7,8], and bone [2,9], as well as brain tumors and meninges [10,11,12]. Among its important biomedical applications are structural studies of fibrosis and pathology-related bone remodeling [13]. This rapidly developing field has important potential for the development of novel quantitative diagnostic approaches and for studies of pathological mechanisms in bone marrow diseases. Despite a handful of highly important pioneering studies [3,14], SHG-based analysis of collagen microarchitecture in bone marrow biopsies remains severely underexplored. Because SHG signal generation is highly sensitive to fibrillar collagen organization and packing geometry, the method provides unique opportunities for the quantitative analysis of extracellular matrix microarchitecture in pathological tissue remodeling [15].

The bone marrow microenvironment plays a central role in the development and progression of myeloproliferative neoplasms and other hematological malignancies [16,17,18]. Increasing evidence suggests that tumor-associated vascular, endosteal, and fibrotic niches critically influence tumor progression, stem cell maintenance, inflammatory signaling, and therapy response [16,19]. In particular, fibrosis-associated extracellular matrix remodeling and pathology-associated alterations of trabecular bone architecture represent important but still poorly understood components of the bone marrow tumor niche microenvironment [20,21,22].

Quantitative bone morphometry provides objective measurements of tissue remodeling that complement conventional histopathological evaluation not only in bone marrow pathology but also across a broad spectrum of bone diseases. In particular, morphometric analysis enables the quantitative assessment of bone formation and resorption, trabecular organization, and fibrosis progression, thereby facilitating objective comparison between specimens, monitoring of disease progression, evaluation of treatment-induced changes, and investigation of bone remodeling mechanisms [23,24,25].

In recent years, SHG imaging has been increasingly applied to fibrosis assessment in bone marrow biopsies, including automated grading approaches for myelofibrosis [3]. However, most studies have primarily focused on fibrotic collagen accumulation, while the collagen architecture of trabecular bone within trephine biopsy samples remains largely unexplored. This limitation is notable because myeloproliferative neoplasms are frequently associated not only with fibrosis, but also with profound alterations in bone remodeling and osteosclerotic microarchitecture [20,21,26]. Given the high sensitivity of SHG microscopy to fibrillar collagen organization [1,2], the method may provide unique opportunities for quantitative analysis of both fibrosis and bone structure within the same biopsy specimen.

Beyond primary myelofibrosis, alterations in collagen microarchitecture also occur in a variety of bone pathologies associated with extracellular matrix remodeling, including inflammatory conditions, impaired bone repair, and radiation-induced bone damage. SHG microscopy has already been applied to investigate collagen organization in several of these settings, demonstrating its potential for the quantitative assessment of pathological collagen remodeling [27,28].

Recent advances in machine learning-assisted image analysis further expand the diagnostic and analytical potential of SHG microscopy for collagen-rich tissues. In particular, shallow learning approaches integrated into user-friendly image analysis environments provide important advantages for exploratory histopathological studies because they require minimal training datasets while allowing rapid generation of reproducible segmentation workflows. An additional advantage of shallow learning approaches lies in their explainability. In contrast to many deep learning models that often operate as “black boxes”, shallow learning enables the user to directly control the annotation process, understand how the classifier is trained, and iteratively refine the segmentation strategy. Explainable AI is attracting increasing interest in biomedical image analysis and clinical pathology, where transparency and reproducibility are considered important prerequisites for future clinical implementation [29,30]. We recently demonstrated the applicability of the LabKit shallow learning framework for SHG image analysis of collagen fiber network microarchitecture [12]. Another line of evidence suggests that LabKit shallow learning annotation strategies critically influence segmentation outcomes and downstream morphometric parameters in biomedical image analysis [31].

A growing body of literature has further emphasized that the amount, quality, and selection strategy of annotated training data are key determinants of segmentation performance in biomedical image analysis. Accordingly, considerable efforts are currently directed toward annotation-efficient learning strategies aimed at reducing the expert annotation burden while preserving segmentation accuracy [32,33].

Here, we applied high-resolution SHG microscopy combined with machine learning-assisted morphometry to quantitatively analyze collagen microarchitecture in bone marrow trephine biopsies from patients with primary myelofibrosis.

We hypothesized that the distinct morphological organization of structured trabecular bone, unstructured trabecular bone, and fibrosis is reflected by differences in collagen SHG-derived microarchitecture that can be quantified using machine learning-assisted morphometric analysis. Accordingly, the aims of this descriptive proof-of-concept study were (i) to develop and evaluate a reproducible workflow for quantitative SHG morphometry and (ii) to identify quantitative collagen microarchitectural descriptors capable of distinguishing these tissue-specific collagen organizations and potentially applicable to the quantitative characterization of collagen remodeling in bone marrow pathology. We further evaluated the efficiency of different annotation strategies for robust segmentation of the collagen SHG images.

## 2. Results

### 2.1. High-Resolution SHG Imaging of Trephine Biopsy Samples

**Figure 1:**
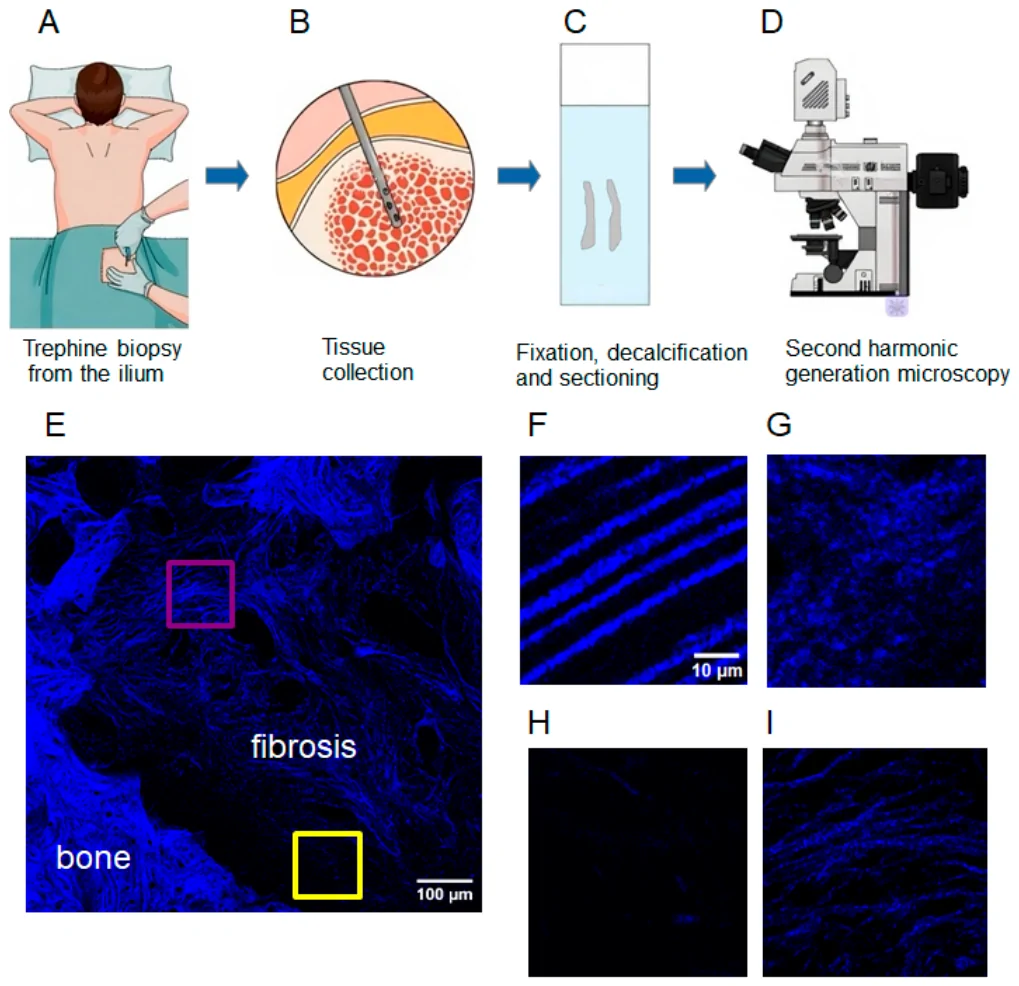
Second harmonic generation microscopy of bone marrow. Trephine biopsy of ilium (**A**) was performed to collect bone marrow samples (**B**), subjected to decalcification, sectioning (**C**) and SHG microscopy with 800 nm excitation (**D**). Images of 4096 × 4096 pixel size were collected (**E**) at pixel size 200 nm. Structured bone (**F**), unstructured bone (**G**), sparse fibrosis (**H**), (yellow squared area in (**E**)), intense fibrosis (**I**), (purple squared area in (**E**)) were observed. Structured trabecular bone is characterized by a prominent parallel striped SHG pattern, whereas unstructured trabecular bone lacks this regular organization. Fibrotic collagen is represented by SHG-positive fibers located outside the trabecular bone.

We first aimed to obtain high-resolution SHG images of bone and fibrosis in trephine biopsy samples from human patients diagnosed with primary myelofibrosis (Figure 1A–E). While SHG imaging is generally considered to be comparable to confocal microscopy in terms of the spatial resolution limit [34,35], we encountered a major limitation in image resolution due to sample photodamage when using high-NA objectives (20×, 63×). The highest spatial resolution we could obtain was at a pixel size of 200 nm using a 10×/0.4 objective (Figure 1). We collected 4096 × 4096 pixel images containing bone and myeloid tissue (Figure 1E). We observed high-resolution high-contrast images of collagen fibers both within the bone and in fibrotic areas (Figure 1E). The bone images contained both highly structured areas of parallelly oriented fibers, which we refer to here as structured bone (Figure 1F), and relatively poorly structured areas (unstructured bone) (Figure 1G). The extent of fibrosis varied from mild cases with sparse fibers (Figure 1E,H) to extensive collagen networks spanning large areas of the sections (Figure 1E,I).

### 2.2. Creation of LabKit Classifiers

**Figure 2:**
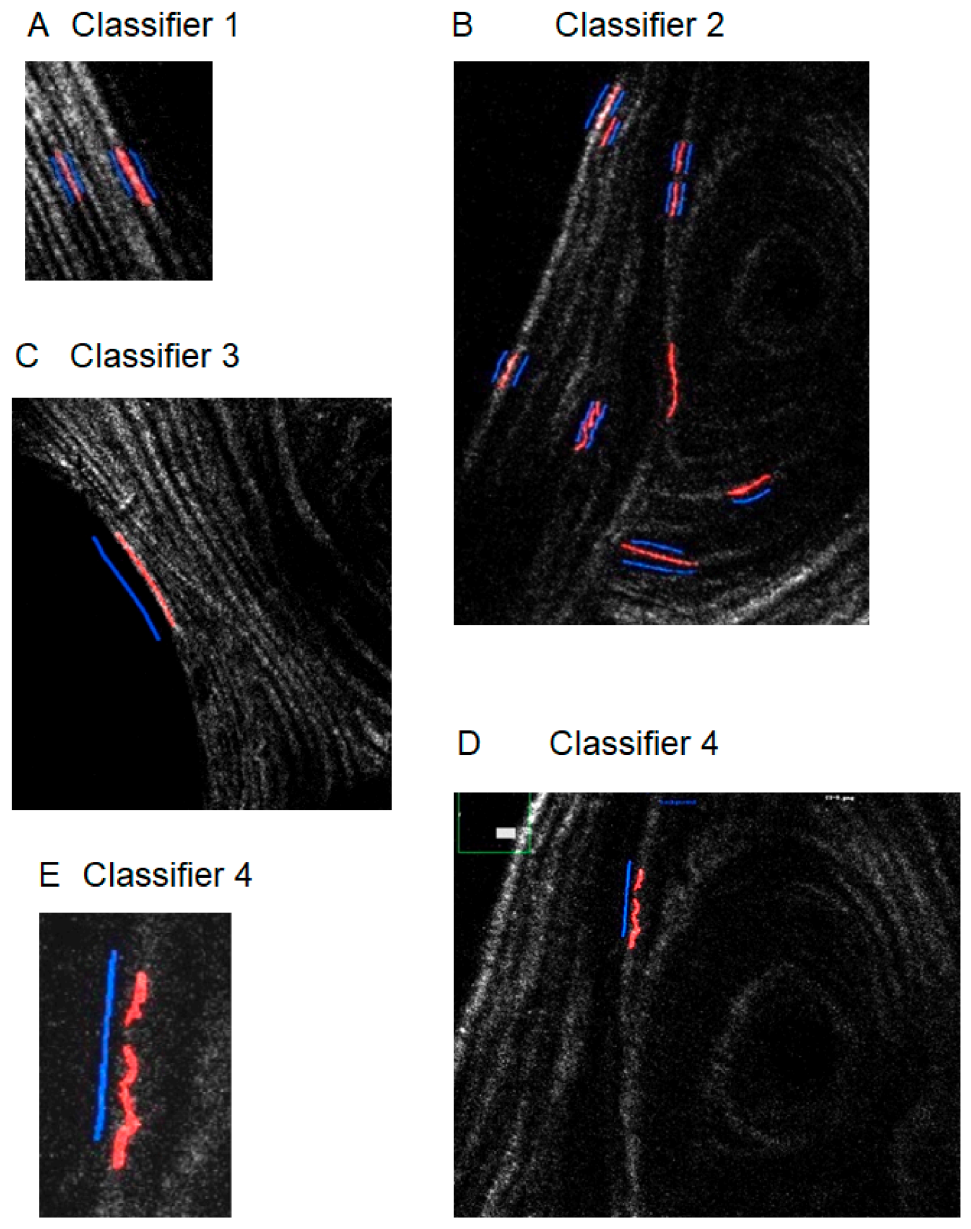
LabKit shallow learning classifier creation. Several classifiers were produced implementing various annotation strategies in terms of SHG intensity, fiber straightness/tortuosity, fiber number, and the number of background lines. Foreground (red) and background (blue) annotation is shown for classifiers 1–4 (**A**–**E**).

**Figure 3:**
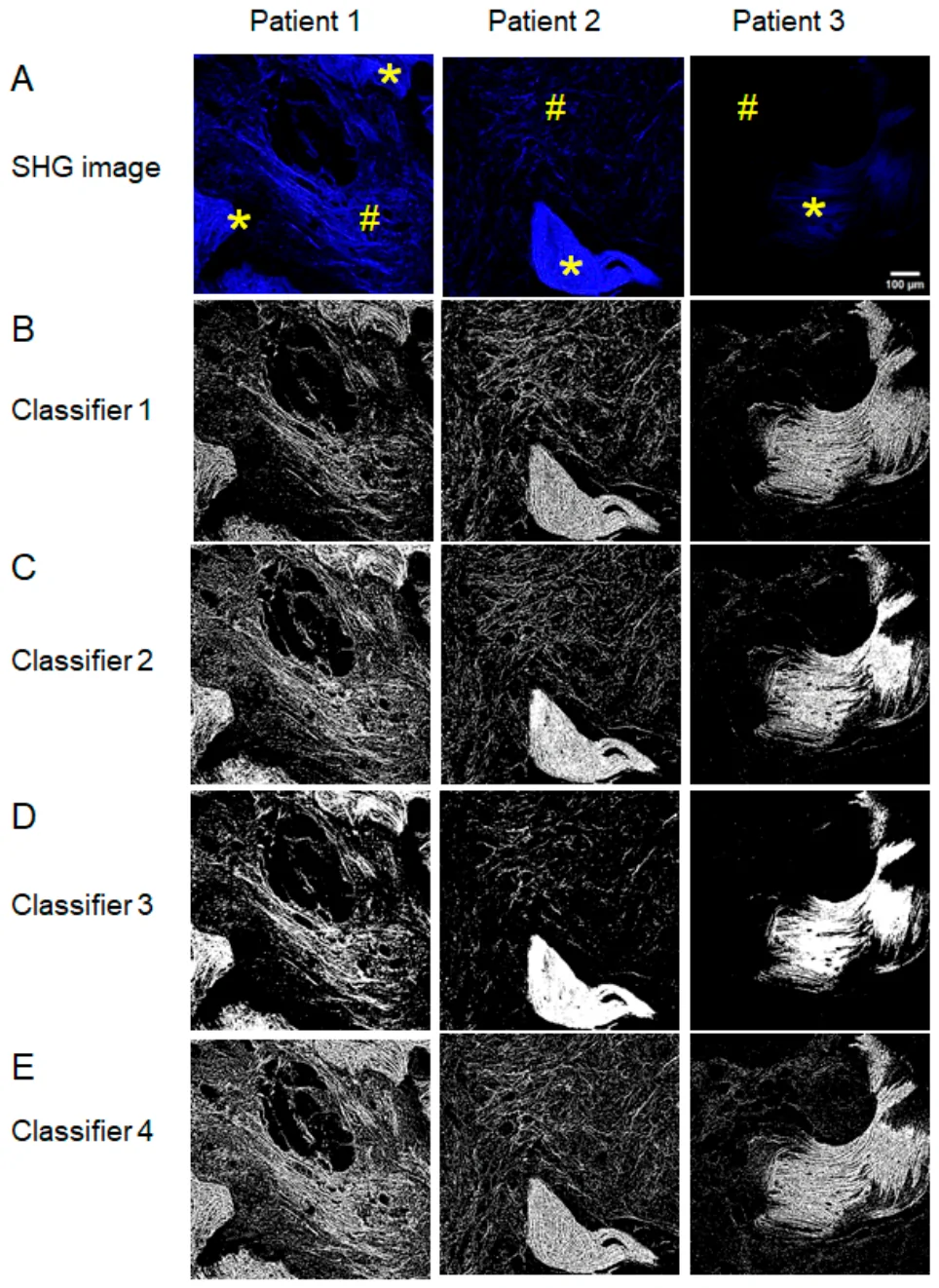
LabKit shallow learning classifier testing. Representative raw SHG images containing bone (marked with an asterisk) and fibrosis (marked with a hashtag) are shown for three patients (**A**). LabKit segmentation results are shown for classifiers 1–4 (**B**–**E**). The same images were segmented independently with each classifier to enable direct visual comparison of segmentation performance.

We then asked whether the recently developed shallow learning-based image segmentation algorithm LabKit [36] could be used for the segmentation of bone and fibrosis SHG images from trephine sections. We created several LabKit classifiers to assess how robustly the algorithm performs on fibers of various sizes, shapes, and SHG intensity levels (Figure 2 and Figure 3). One crucial advantage of the LabKit shallow learning method is that it allows a skilled researcher to create a single classifier in less than one minute, which can then be applied to multiple images.

We tried a broad repertoire of various annotation strategies to elaborate efficient shallow learning-based collagen SHG image segmentation (Figure 2). The foreground sampling varied with respect to the SHG intensity, fiber straightness/tortuosity, and the number of fibers. For the creation of classifier 1, two fibers were selected: one exhibiting medium SHG signal intensity and the other showing SHG signal intensity close to the maximum observed values. For classifier 2, nine representative fibers distributed across the low-to-medium SHG intensity range were selected. Background regions were simultaneously annotated alongside the fibers. For classifier 3, a single fiber exhibiting the highest SHG signal intensity was selected, and a corresponding background region was annotated in parallel. For classifier 4, a low-intensity fiber segment was selected. The foreground annotation line was curved and discontinuous. A single straight line was used to annotate the background parallel to the foreground annotation. For classifier 5, five fiber segments were selected, three of which belonged to the same fiber. The selected segments exhibited medium SHG signal intensity. Background regions were annotated on both sides of the selected segments using straight annotation lines. For classifier 6, two low-intensity segments of the same fiber were selected, and background regions were annotated on both sides in parallel.

### 2.3. Comparison of Classifiers

Comparison of the resulting segmentation masks demonstrated that classifiers 1 and 2 segmented structures exhibiting medium SHG signal intensity with comparable performance (Figure 3). However, classifier 2 produced a more detailed segmentation pattern; nevertheless, in regions of high SHG signal intensity, adjacent structures frequently merged into larger connected objects.

Classifier 3 segmented primarily structures exhibiting high SHG signal intensity, whereas structures with low SHG signal intensity were largely excluded from the segmentation results. In addition, high-intensity structures were frequently segmented as single merged objects.

Classifier 4 segmented structures across the full range of SHG signal intensities with consistently high performance, while minimizing the formation of merged objects in high-intensity regions.

Classifiers 5 and 6 showed similar performance when segmenting structures exhibiting low-to-medium SHG signal intensity.

To further validate this qualitative assessment, two experts independently evaluated the segmentation results obtained with classifiers 1–6. Based on a consensus assessment, three classifiers (1, 4, and 6) were identified as producing the most accurate segmentation and were selected for further quantitative comparison.

Segmentation of the relatively weak SHG signal from thin collagen fibers in fibrosis can be considered the most challenging segmentation task. Therefore, quantitative validation was performed using fibrosis images, providing the most stringent test of segmentation performance. Classifier 4 consistently outperformed classifier 6 according to both Dice and IoU measurements (Figure S2). Compared with classifier 1, classifier 4 showed a moderate improvement in IoU, corresponding to a Cohen’s dz of 0.688 (Figure S2).

Based on the comparison of segmentation results obtained for structured bone, unstructured bone, and fibrotic tissue, classifier 4 demonstrated the best overall performance. It provided the most accurate segmentation across all SHG signal intensity ranges, avoided artificial merging of bright structures, and preserved low-intensity fibers that were frequently missed by other classifiers, thereby generating the most detailed morphological representation (Figure 3).

### 2.4. Training Rules for Successful Classifiers

Systematic comparison of the foreground and background annotation strategies (red and blue annotation lines in Figure 2) enabled us to formulate a set of practical guidelines for training efficient LabKit classifiers for bone marrow SHG image segmentation. The proposed rules are as follows. (1) Foreground and background annotation lines should be placed in close proximity to one another. (2) Background annotations should preferably be drawn on both sides of the foreground annotation. If only one background line is used, it should be positioned in a region exhibiting the highest possible intensity contrast relative to the foreground. (3) Foreground annotations should follow continuous collagen fibrils rather than interrupted signal fragments and should include both high- and low-intensity pixels whenever possible. (4) The foreground annotation should span the full width of the collagen fibril rather than only its central axis.

**Figure 4:**
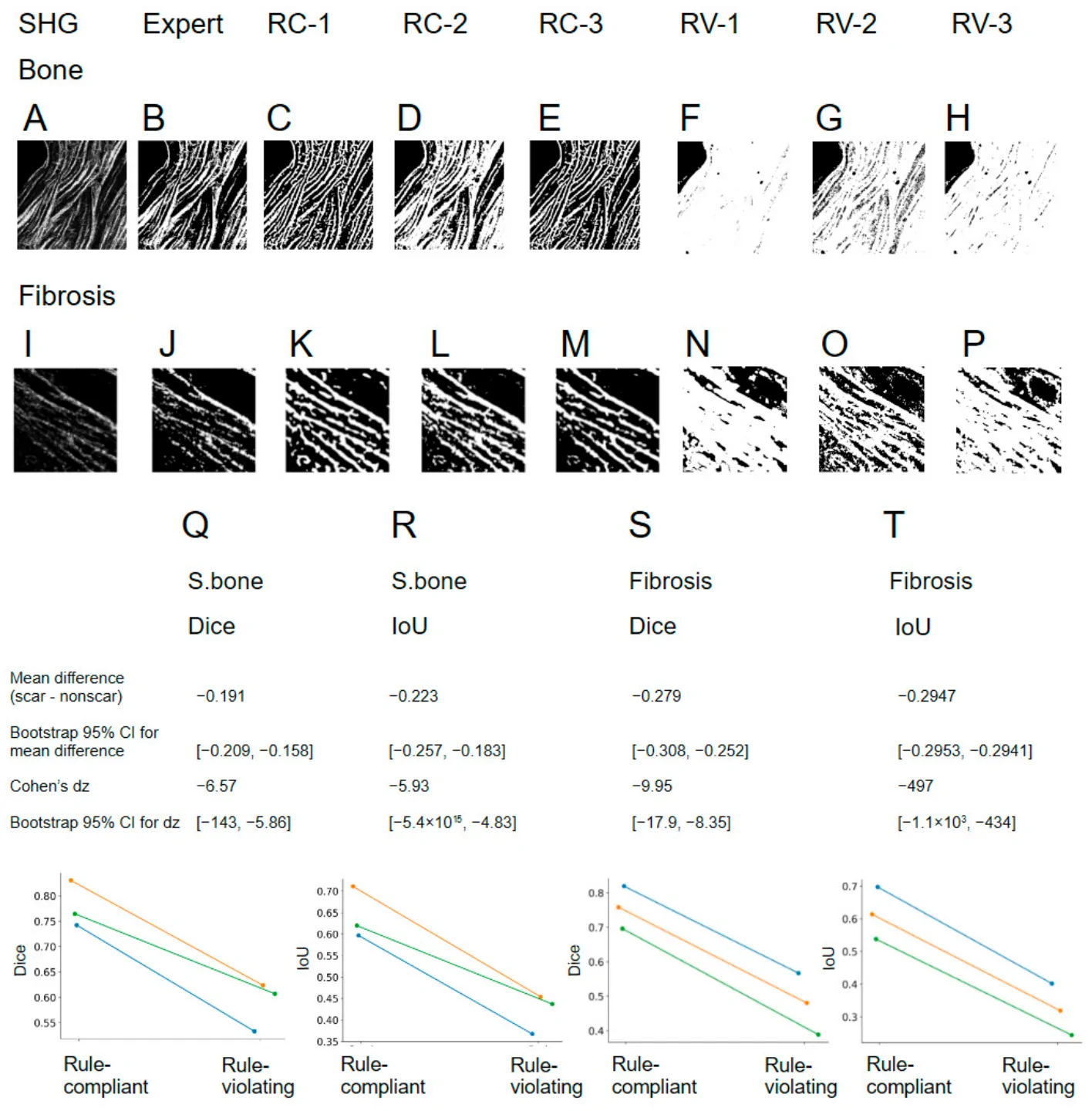
Segmentation performance is affected by training rules for shallow learning. (**A**,**I**)—SHG images of structured bone and fibrosis. (**B**,**J**)—Expert-selected threshold-based reference masks for A, I, with threshold set by a human expert. (**C**–**E**)—Bone segmentation results for rule-compliant classifiers 1–3, respectively. Abbreviations: RC, rule-compliant classifiers; RV, rule-violating classifiers. (**F**–**H**)—Bone segmentation results for rule-violating classifiers 1–3, respectively. (**K**–**M**)—Fibrosis segmentation results for rule-compliant classifiers 1–3, respectively. (**N**–**P**)—Fibrosis segmentation results for rule-violating classifiers 1–3, respectively. Two different match indices—the Dice coefficient and Intersection over Union (IoU)—were calculated between the expert- and LabKit-derived masks, and the values were averaged across the three rule-compliant and three rule-violating classifiers for structured trabecular bone (**Q**,**R**) and fibrosis (**S**,**T**). Data are presented as averages per patient for 3 patients (N = 3). The dataset included 3 ROIs from each patient, for a total of 9 ROIs for structured bone and 9 ROIs for fibrosis. Statistical analysis was performed on per-patient mean values (N = 3 independent patients; 3 ROIs averaged for each patient). Each colored line represents one patient.

To evaluate these guidelines experimentally, we generated an independent set of six additional classifiers: three trained according to the proposed annotation rules and three intentionally trained in violation of these rules (Figure S1). The segmentation accuracy was assessed by calculating two overlap metrics, the Dice coefficient and Intersection over Union (IoU), between expert-selected threshold-based reference masks and the corresponding LabKit-derived segmentation masks. Across a dataset comprising nine ROIs of structured trabecular bone and nine ROIs of fibrosis obtained from three independent patients, classifiers trained according to the proposed annotation guidelines consistently achieved higher Dice and IoU values than classifiers deliberately trained in violation of these guidelines (Figure 4).

### 2.5. Fiji Pipelines with Embedded Machine Learning

The LabKit plugin was originally developed to facilitate integration into reproducible Fiji workflows and high-throughput image analysis pipelines [36]. Taking advantage of these capabilities, we developed two Fiji-based workflows for semi-automated and automated analysis of bone marrow SHG images.

**Figure 5:**
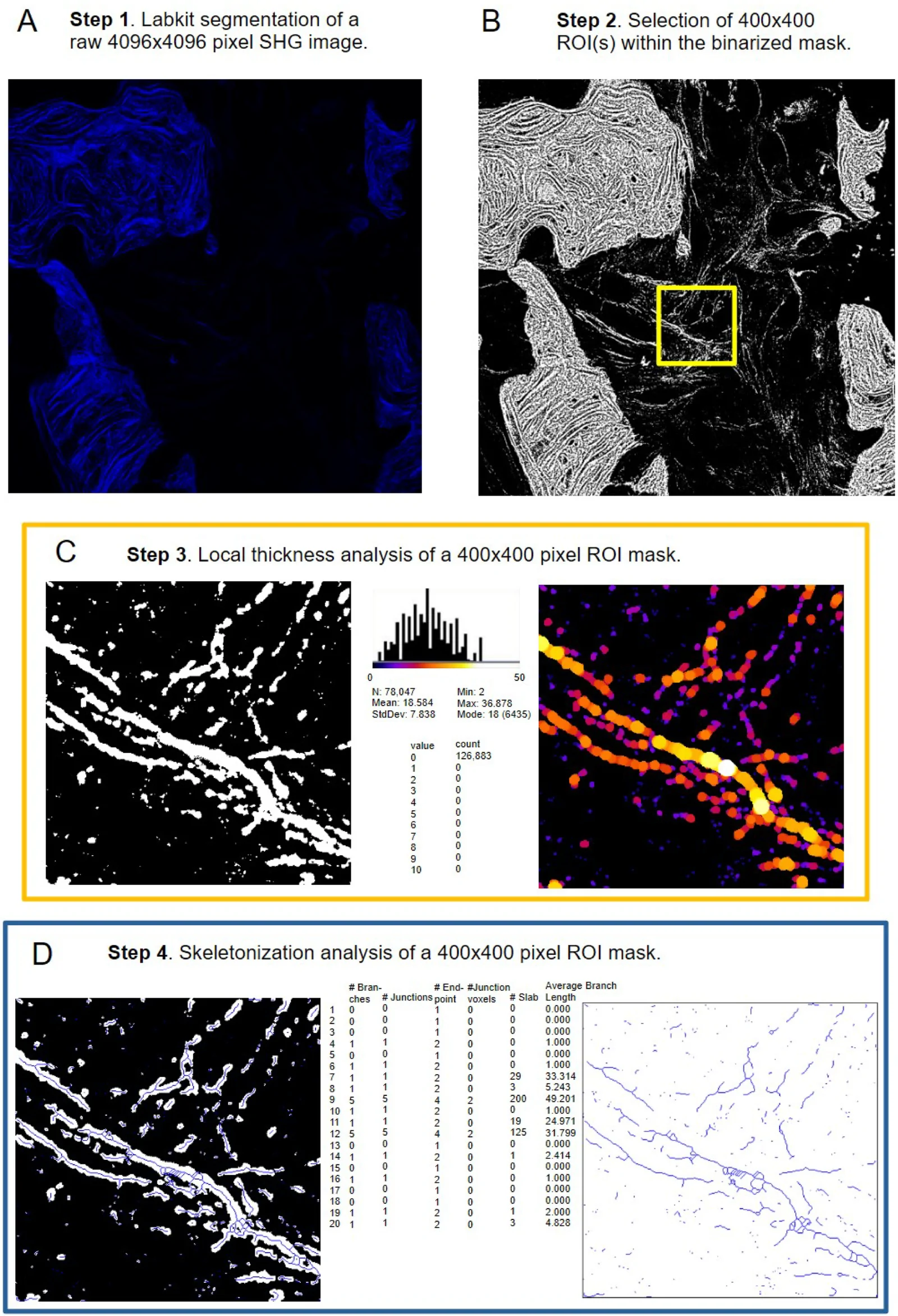
Schematic representation of the pipeline 1. LabKit segmentation (**A**) is followed by ROI selection (**B**), local thickness (**C**) and skeleton (**D**) analysis. The workflow includes LabKit segmentation of the original 4096 × 4096-pixel SHG image (**A**), manual selection of 400 × 400-pixel ROIs within the resulting binary mask (**B**), local thickness analysis for quantification of collagen fiber width (**C**), and skeletonization for extraction of collagen network branching parameters (**D**). In the pseudocolor map in (**C**), warmer colors indicate higher local collagen fiber thickness, whereas cooler colors correspond to thinner fibers.

Pipeline 1 operates on 4096 × 4096 pixel images. The workflow begins with SHG image segmentation using a pre-trained LabKit classifier, which generates a binary mask (Figure 5). The center of each region of interest (ROI) is then selected manually using the PointPicker tool. Each ROI measures 400 × 400 pixels, and multiple ROIs may be selected during a single pipeline run.

The resulting binary masks are subsequently subjected to two complementary morphometric analyses using Fiji plugins: (1) local thickness analysis, which generates a fiber width histogram and a pseudocolor thickness map, and (2) skeleton analysis, which extracts quantitative parameters describing collagen network branching geometry.

All morphometric measurements are automatically exported to output files for subsequent statistical analysis. Additional image-processing operations and morphometric measurements can be incorporated into the workflow with minimal modifications. Compared with manual image processing, this workflow increases the analysis speed by approximately 8.5-fold while substantially reducing operator intervention throughout the analysis procedure. Pipeline 2 processes a predefined set of ROIs and performs LabKit-based segmentation followed by the same morphometric analysis steps as in Pipeline 1.

### 2.6. Quantitative Analysis of the Bone and Fibrosis Collagen Microstructure

After identifying classifier 4 as the optimal classifier, we used it for the quantitative analysis of the bone and fibrosis collagen microstructures. Binarized masks of 820 × 820 μm SHG images were obtained using classifier 4 (Figure 5A). We then selected ROIs of 80 × 80 μm size for fibrosis and structured bone and unstructured bone areas of the trephine sections (Figure 5B). Ten ROIs of each tissue type per patient were selected from the 3 patients. The local thickness and skeleton plugins were applied in Fiji to retrieve the quantitative parameters of the collagen microstructure.

#### 2.6.1. Collagen Fiber Width

**Figure 6:**
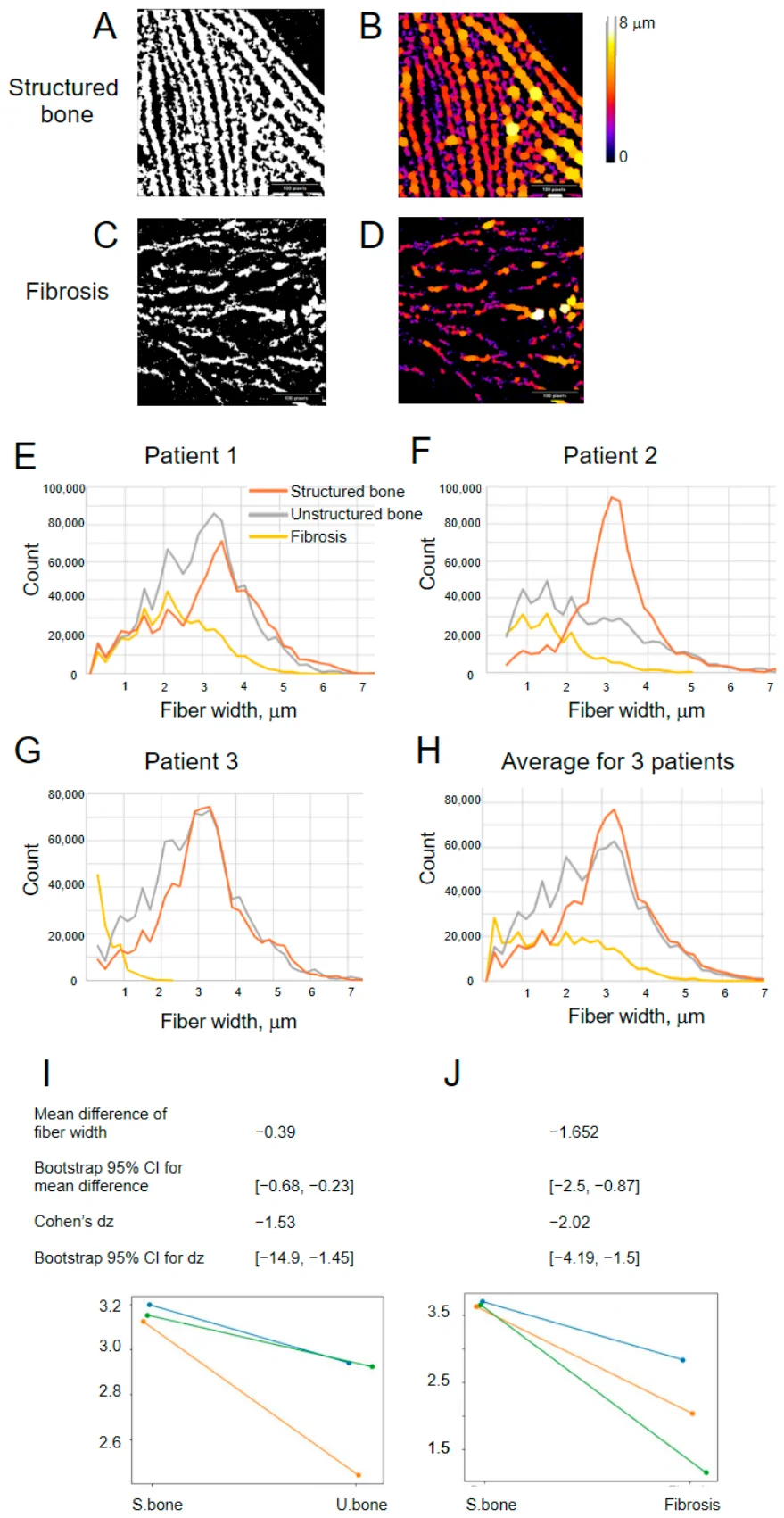
Collagen fiber width quantification in bone marrow SHG images. Following Labkit segmentation of the raw SHG images with classifier 4, the binarized masks of bone (**A**) and fibrosis (**C**) were processed with the local thickness algorithm in Fiji ((**B**,**D**), respectively). The pseudocolor scale shown in panel (**B**) represents local collagen fiber thickness and is identical for panels (**B**) and (**D**). Warmer colors correspond to thicker fibers. Histograms of the fiber local thickness (µm) measurements are shown for structured bone, unstructured bone and fibrosis (averaged over 10 ROIs in each case) for patients 1–3 ((**E**–**G**), respectively) and averaged for 3 patients (**H**). Bootstrap analysis showed strong difference in mean fiber thickness between structured bone and fibrosis (**I**), as well as between structured and unstructured bone (**J**). The local thickness pseudocolor code in (**B**) is valid for (**B**,**D**). Statistical analysis was performed on per-patient mean values (N = 3 independent patients; 10 ROIs averaged per tissue type for each patient). Each colored line represents one patient.

We observed remarkable differences in the fiber width distribution between the structured trabecular bone, unstructured trabecular bone and fibrosis (Figure 6). Across all three patients, collagen fibers were consistently thinner in fibrosis than in trabecular bone (Figure 6H,J). Next, the fiber width distribution is clearly shifted to the left for the unstructured bone compared with the structured bone for all three patients (Figure 6H,I). The structured trabecular bone demonstrates a consistent profile of the fiber thickness distribution for all three patients with the common maximum at about 3.2 μm (Figure 6E–H). By contrast, both fibrosis and unstructured bone exhibit remarkable variability in fiber thickness, suggesting different states of the pathologic process.

#### 2.6.2. The Collagen Network Geometry

- **Gross analysis.**

**Figure 7:**
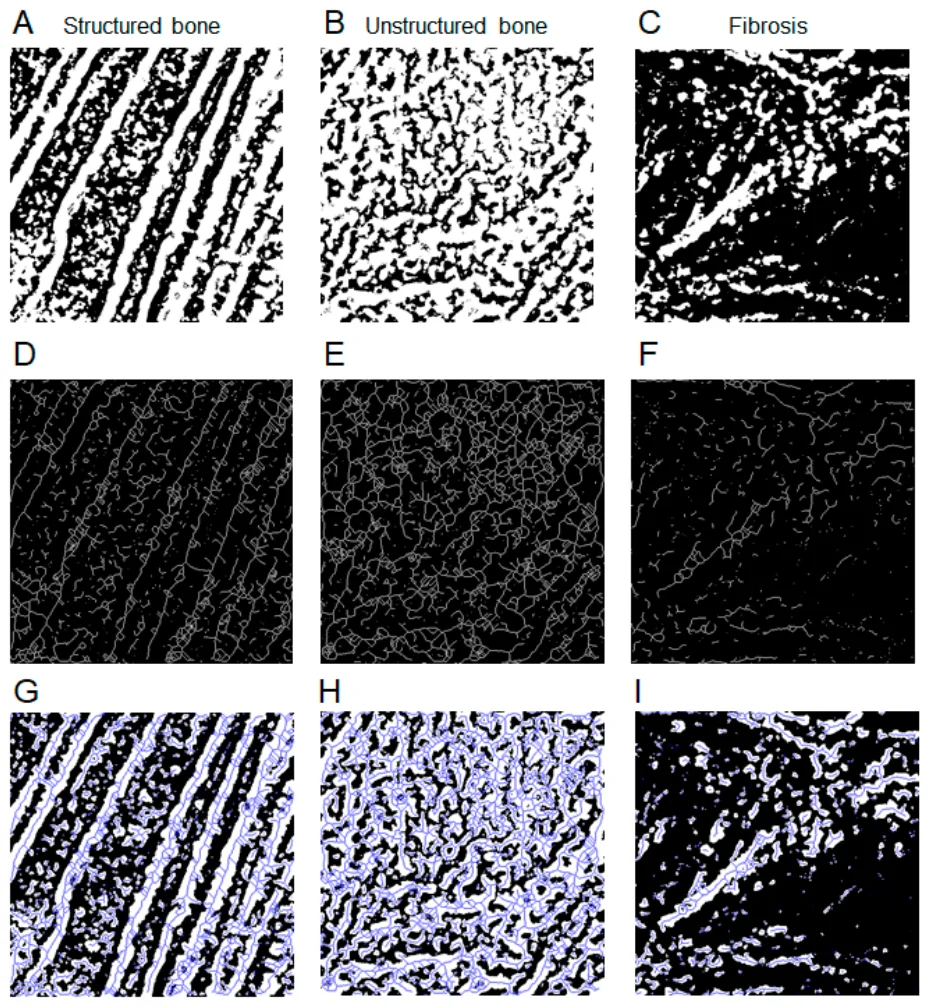
Skeletonization of LabKit-segmented SHG bone marrow images. Binarized masks of structured bone (**A**), unstructured bone (**B**) and fibrosis (**C**) were processed with the skeleton plugin in Fiji. The resulting skeleton network architecture (**D**–**F**) exhibits clear differences among the three types of SHG images. (**G**–**I**) The skeleton overlay on SHG masks.

**Figure 8:**
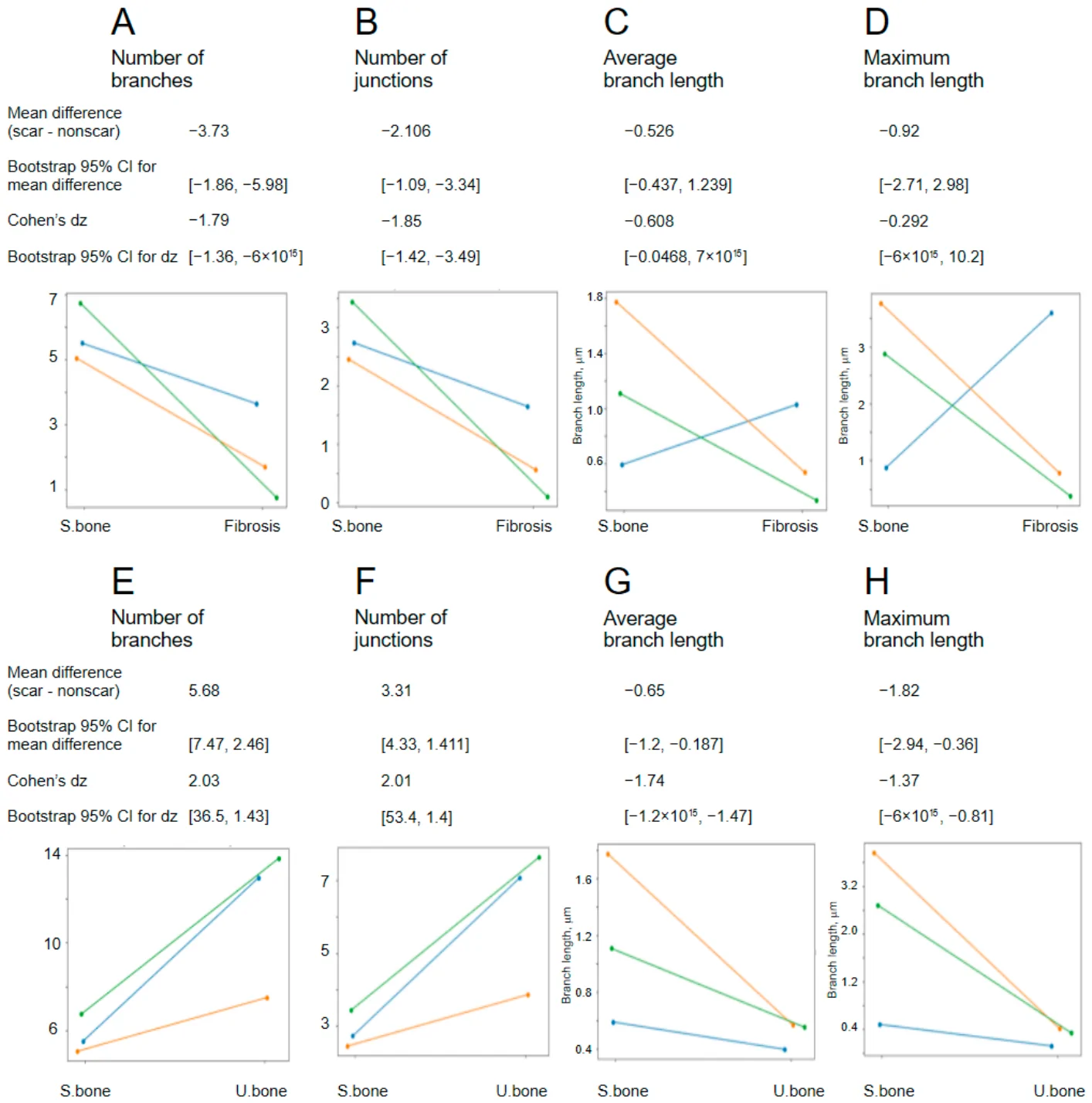
Collagen fiber network skeletonization-derived parameters reveal the network geometry difference between structured bone, unstructured bone and fibrosis. Branching is consistently higher for the structured bone as compared to fibrosis (**A**,**B**) and consistently higher for unstructured bone as compared to the structured bone (**E**,**F**). Average and maximum branch length do not exhibit a consistent difference between structured bone and fibrosis (**C**,**D**), but those parameters show consistently higher values for structured bone as compared to unstructured bone (**G**,**H**). Data are presented as an average over objects of all sizes and branching complexity levels. Statistical analysis was performed on per-patient mean values (N = 3 independent patients; 10 ROIs averaged per tissue type for each patient). Each colored line represents one patient.

**Figure 9:**
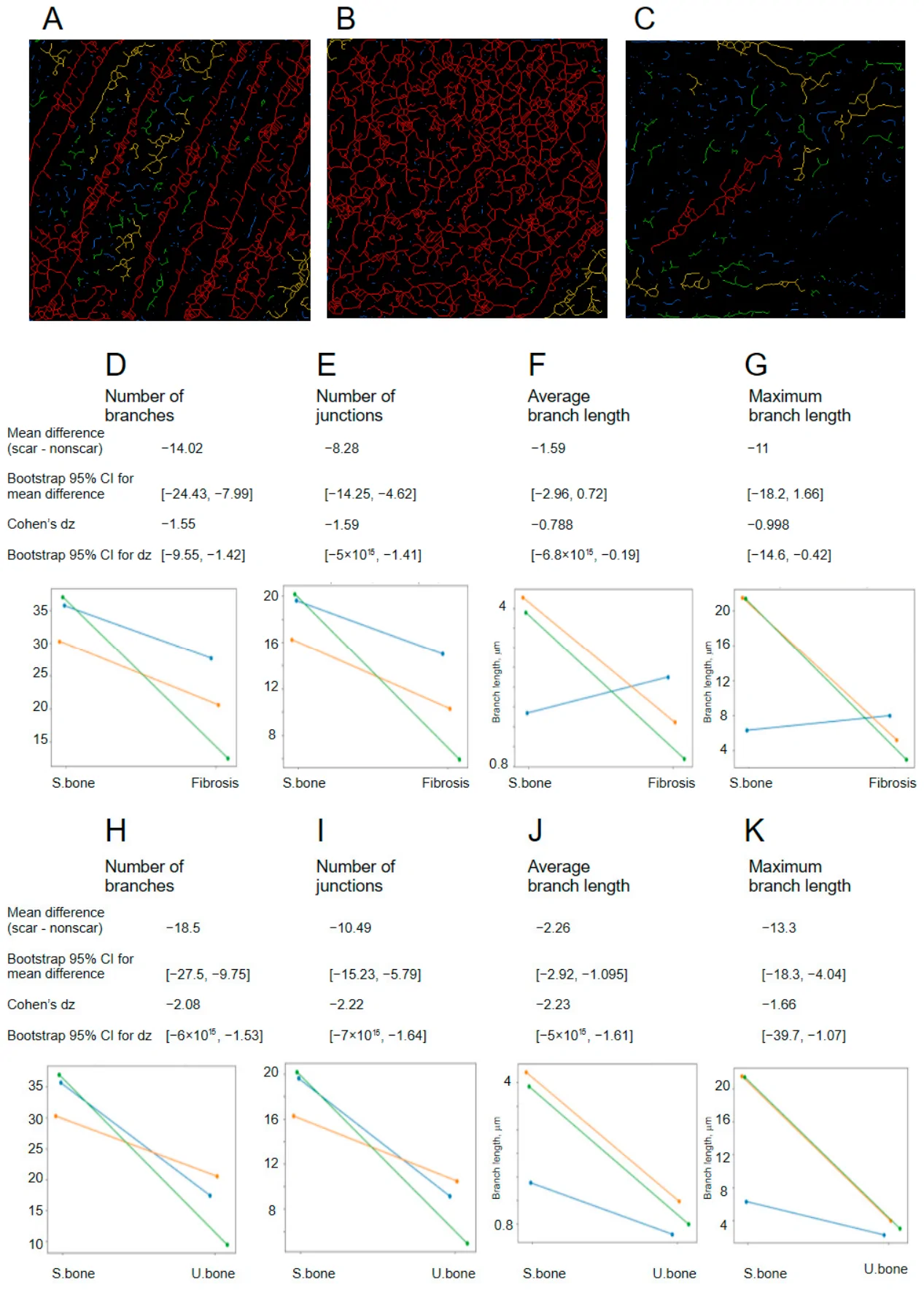
Differential analysis of the collagen fiber network skeletonization-derived parameters based on the network branching complexity. (**A**–**C**) The skeletonization-derived objects were divided into four bins based on the number of branches: 0–1 branches (color-coded in blue), 2–10 (green), 11–100 (yellow), over 100 branches (red). The same representative masks for structured bone (**A**), unstructured bone (**B**) and fibrosis (**C**) are shown as in Figure 7. (**D**–**K**). Bootstrap analysis for the bin of 11–100 branches. Branching is consistently higher for the structured bone as compared both to fibrosis (**D**,**E**) and to the unstructured bone (**H**,**I**). Average and maximum branch length do not exhibit consistent difference between structured bone and fibrosis (**F**,**G**), but those parameters show consistently higher values for structured bone compared with unstructured bone (**J**,**K**). Statistical analysis was performed on per-patient mean values (N = 3 independent patients; 10 ROIs averaged per tissue type for each patient). Each colored line in (**D**–**K**) represents one patient.

Using the skeleton plugin in Fiji we retrieved several quantitative parameters of the trabecular bone and fibrosis collagen network, including the number of branches, the number of joint points, the mean and maximum branch length, etc. (Figure 7 and Figure 8). The plugin detects both small unbranched objects and networks of various size and complexity (Figure 9A–C). We first compared the average values for the geometry parameters of all objects between structured bone, unstructured bone and fibrosis (Figure 8). Across the three patients, structured trabecular bone consistently exhibited more branching than fibrosis with no clear difference in the branch length (Figure 8A–D). In turn, the unstructured bone was more branched than the structured bone and exhibited systematically shorter branches (Figure 8E–H).

- **Branching-based analysis.**

To obtain a more detailed characterization of collagen network geometry, we performed an additional branching-based analysis of the skeletonization-derived objects. The analysis was performed in the following steps. First, binary masks of structured trabecular bone, unstructured trabecular bone, and fibrosis were skeletonized using the Fiji skeleton plugin. The resulting dataset contained objects with a broad range of geometries, from isolated single-pixel objects and short unbranched segments to large highly branched collagen networks. Second, because averaging morphometric parameters across this heterogeneous population could obscure differences between tissue types, the detected skeleton objects were divided into four groups according to their number of branches: 0–1, 2–10, 11–100, and >100 branches (Figure 9A–C). Third, within each branch-number group, skeletonization-derived morphometric parameters were compared between structured trabecular bone, unstructured trabecular bone, and fibrosis. Statistical comparisons were performed using per-patient mean values, with 10 ROIs averaged for each tissue type in each patient.

For objects containing 0–1 branches, highly consistent differences between structured and unstructured trabecular bone were observed in the branch number and branch length across all three patients, whereas no clear difference was detected between structured bone and fibrosis.

For objects containing 2–10 branches, structured bone and fibrosis differed most clearly in the maximum branch length (mean difference of 5 μm). Structured and unstructured bone also differed in the mean and maximum branch length.

For objects containing 11–100 branches, structured bone consistently exhibited a higher number of branches than both fibrosis and unstructured bone. In addition, the branch length was consistently higher in structured bone than in unstructured bone (Figure 9D–K).

For objects containing >100 branches, representing the largest and most highly connected collagen networks, consistent differences were observed between structured bone and fibrosis and between structured and unstructured bone in both branching and branch-length parameters.

#### 2.6.3. Collagen Orientation

**Figure 10:**
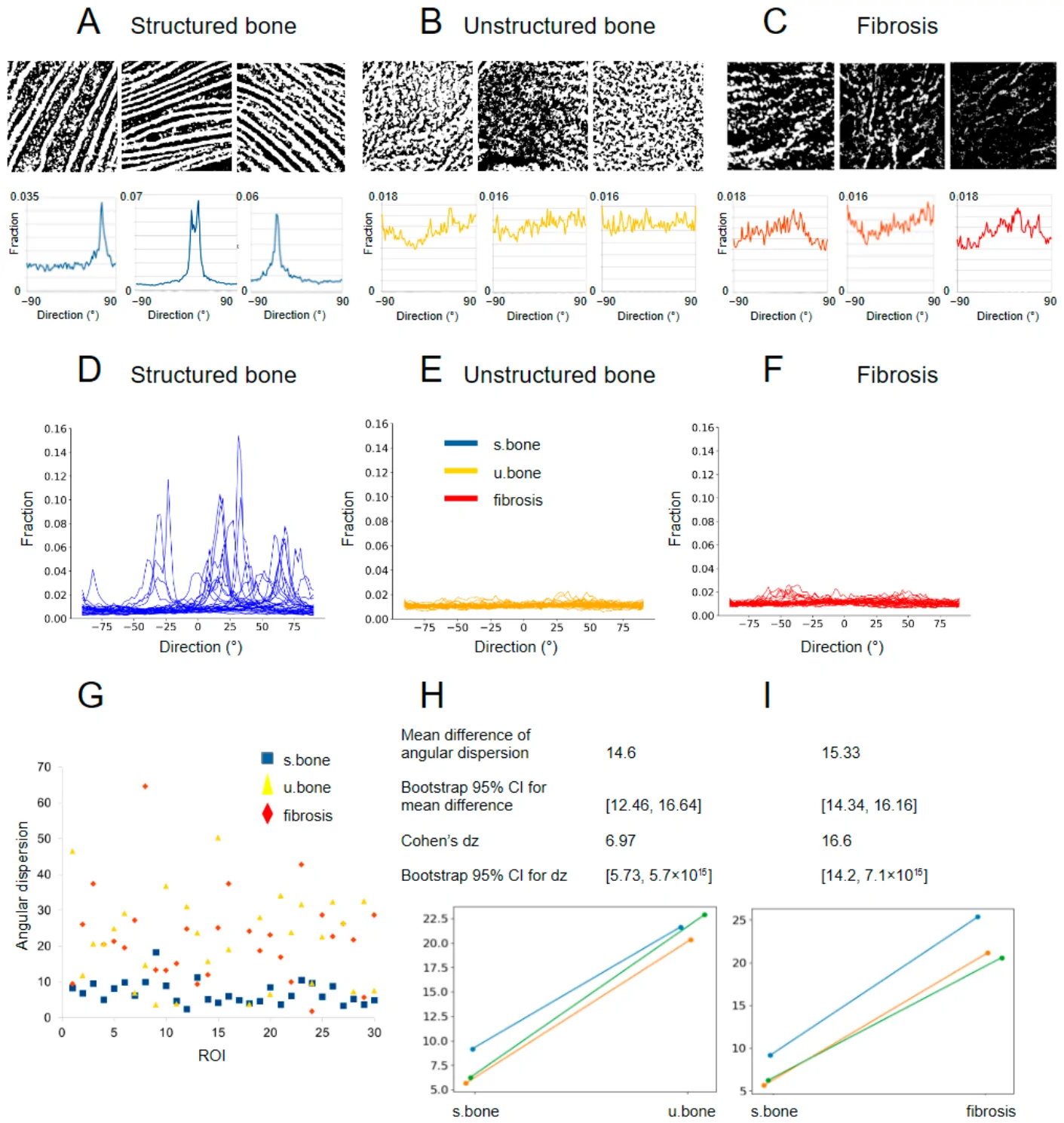
Angular dispersion of collagen orientation reveals quantitative differences between structured trabecular bone, unstructured trabecular bone, and fibrosis in SHG images. (**A**–**C**) Representative images from a single patient are shown for structured trabecular bone (**A**), unstructured trabecular bone (**B**), and fibrosis (**C**). Corresponding orientation histograms are shown below each image. Note the different y-axis scales across panels. Orientation distributions were obtained using the Fiji directionality plugin (Fourier components method). (**D**–**F**) Orientation histograms for individual ROIs of structured trabecular bone (**D**), unstructured trabecular bone (**E**), and fibrosis (**F**). Data are shown for 30 ROIs per tissue type (10 ROIs per patient per tissue type across 3 patients). (**G**) Single-ROI angular dispersion scatter plot for the same dataset as in (**D**–**F**). (**H**,**I**) Structured trabecular bone exhibits systematically lower angular dispersion compared to unstructured trabecular bone (**H**) and fibrosis (**I**). Statistical analysis was performed on per-patient mean values (N = 3 independent patients; 10 ROIs averaged per tissue type for each patient). Each colored line in (**H**,**I**) represents one patient.

Because SHG signal intensity depends on the orientation of non-centrosymmetric collagen fibrils relative to the optical axis and polarization of the imaging system [37], we next examined collagen orientation in the three tissue types using the Fiji directionality plugin (Fourier components method). Structured trabecular bone exhibited pronounced preferred fiber orientations in all three patients, as demonstrated by distinct peaks in the pooled orientation histograms (Figure S3A). In contrast, orientation distributions in unstructured trabecular bone and fibrosis were considerably broader and generally lacked pronounced dominant directions (Figure S3B,C). Interestingly, although the three patients displayed distinct orientation profiles (Figure S3A), the local thickness histograms showed highly similar peak positions for structured bone across patients (Figure 6E–G), indicating that the observed collagen thickness measurements are largely independent of the overall fiber orientation. To quantify the observed differences in orientation distributions, we compared the angular dispersion values obtained from the directionality plugin. All three patients consistently exhibited lower angular dispersion and stronger directional alignment in structured trabecular bone than in unstructured trabecular bone or fibrosis (Figure 10A–I). By contrast, both unstructured trabecular bone and fibrosis showed substantially broader orientation distributions, reflecting a more isotropic collagen organization (Figure 10).

Together, these findings indicate that angular dispersion provides an additional quantitative descriptor of collagen microarchitecture, complementing local thickness and skeleton-based morphometric parameters for the SHG analysis of bone marrow collagen.

## 3. Discussion

The extracellular matrix is increasingly recognized as an active component of the bone marrow niche rather than a passive scaffold. Collagen organization influences tissue mechanics, cell adhesion, migration, and signaling, thereby contributing to the regulation of hematopoietic and neoplastic cell behavior. The present exploratory study consistently identified distinct collagen microarchitectural patterns for structured trabecular bone, unstructured trabecular bone and fibrosis across the three independent patients examined. The approach described here suggests that collagen microarchitecture may provide a quantitative structural descriptor of bone marrow niches, thereby adding a new dimension to diagnostics and mechanistic studies of bone marrow pathologies. Indeed, in tumors, collagen alignment, bundling, and fiber organization influence cancer cell migration, invasion, mechanotransduction, immune cell infiltration, and therapy response [38,39,40,41]. Likewise, alterations in the collagen organization in bone are associated with changes in tissue mechanical properties and are increasingly recognized as important contributors to osteoporosis and pathology-associated bone remodeling [9,42,43,44]. From this perspective and taking into account the growing role of quantitative bone morphometry across a broad range of bone pathologies [23,24,25], SHG-based collagen morphometry may complement existing cellular and molecular approaches by providing quantitative structural descriptors of the extracellular matrix component of the bone marrow niche microenvironment. Importantly, the present study represents a descriptive proof-of-concept analysis. Therefore, substantially larger patient cohorts will be required to determine the diagnostic value of the proposed approach, to evaluate its potential for biomarker discovery, and to establish its broader applicability across bone marrow pathologies.

Although the present study was not designed to correlate SHG-derived parameters with established fibrosis grades, the observed differences in collagen microarchitecture suggest that future studies may evaluate whether quantitative morphometric descriptors provide information complementary to current WHO and European consensus fibrosis grading systems [3,19].

While the histopathological assessment of bone marrow fibrosis has traditionally relied on the extent of collagen deposition and fibrosis grading, the present results suggest that SHG imaging may provide several additional quantitative descriptors of collagen organization, including the fiber thickness, branching complexity, and network geometry. These parameters may represent an additional dimension of bone marrow pathology and could potentially expand the repertoire of quantitative tools available for differential diagnostics [13,15]. Future studies may determine whether SHG-derived collagen microarchitectural descriptors differ between primary myelofibrosis, post-polycythemic myelofibrosis, post-essential thrombocythemia myelofibrosis, reactive fibrosis, and other bone marrow pathologies [16,17].

An important methodological aspect of the present study is the use of shallow machine learning-based segmentation with the LabKit platform. While deep learning approaches dominate current biomedical image analysis, shallow learning methods provide substantial practical advantages for exploratory histopathological applications, including rapid classifier generation, low annotation burden, and direct integration into Fiji workflows. Our analysis further demonstrates that the annotation strategy critically influences the segmentation outcome and the derived morphometric parameters. This observation is particularly important for fibrillar structures, where preservation of low-intensity collagen fibers substantially affects the skeleton topology and quantitative branching analysis. More broadly, it aligns with a growing body of literature emphasizing that the annotation strategy itself represents a major determinant of machine learning performance in biomedical image analysis. Recent annotation-efficient approaches aim to reduce the expert workload by optimizing the selection and representativeness of training annotations while maintaining the segmentation quality [32,33].

To our knowledge, shallow learning approaches have not previously been applied to SHG microscopy image segmentation (except for a recent proof-of-concept study on mouse brain meninges in Paveliev et al. [12]). Therefore, the approach described here opens a new instrumental perspective that may be valuable for the broad and rapidly growing field of SHG biomedical imaging. Moreover, a thorough literature search suggests that image segmentation for bone collagen structural analysis and for fibrosis histological analysis has not previously been addressed with shallow learning either, highlighting the above-mentioned methodological advantages of shallow learning for these important fields of medical research.

In contrast to end-to-end deep learning approaches, the present workflow is based on shallow machine learning-assisted segmentation followed by the extraction of biologically interpretable morphometric parameters. As a result, the final quantitative descriptors remain directly linked to observable collagen features such as fiber thickness, branching complexity, branch length, and orientation dispersion. This transparency facilitates biological interpretation, validation by pathologists, and the identification of potential segmentation artifacts. Such explainable morphometric workflows may offer advantages for clinical implementation because the diagnostic decision can be traced back to specific structural features rather than inferred from latent representations of a neural network [29,30].

Interestingly, a conceptually similar trend towards explainable machine learning can be observed in recent SHG studies of myelofibrosis. In their pioneering work, Yu et al. [3] first extracted biologically interpretable collagen descriptors including the fiber width, length, straightness, orientation and fiber count and only subsequently applied a support vector machine classifier for fibrosis grading. Thus, when shallow learning is used for diagnostic classification rather than image segmentation, the decision remains grounded in explicit morphometric features rather than directly inferred from raw image data. This approach provides important perspectives for the development of novel diagnostic strategies based on label-free multiphoton microscopy and quantitative tissue assessment. Such quantitative imaging approaches may be highly beneficial for a broad range of diagnostic and biomedical applications, including but not limited to gastrointestinal stromal tumors, breast cancer, and pancreatic ductal adenocarcinoma [8,45,46].

A substantial part of the present study was devoted to the creation, selection and testing of classifiers that perform efficiently both on the bone and fibrosis images (Figure 2 and Figure 3). As a result, we created classifier 4, which fulfilled these criteria and performed successfully working on images from independent samples and repeated experiments. Importantly, this result enables the use of a unified procedure at all steps of the study, from tissue imaging to image segmentation and subsequent analysis with local thickness and skeletonization algorithms. To further explore the procedure of efficient classifier creation, we investigated whether practical “rules of thumb” could be formulated for the bone marrow SHG image annotation within the LabKit classifier training. We previously addressed the same question for a relatively simple object—confocal images of GFAP-immunostained astrocytes in mouse brain sections in the experimental model of neuroimplantation-induced glial scarring [31]. In the present study, we extended the approach to formulate and test certain rules for training efficient LabKit classifiers on the bone marrow SHG images. By using two different metrics (the Dice coefficient and IoU), we demonstrate that rule-compliant classifiers perform better than rule-violating classifiers both on the structured bone and fibrosis images (Figure 4). This result suggests that the annotation design of efficient shallow learning classifiers is manageable for a range of biomedical applications and could be further studied for expanding the applicability and increasing efficiency of shallow learning tools.

Our results demonstrate that SHG microscopy combined with machine learning-assisted image analysis enables the quantitative characterization of collagen microarchitecture in bone marrow trephine biopsies. Previous SHG studies of bone have described both highly ordered lamellar collagen organization and more disorganized fibrillar arrangements associated with remodeling, repair, or pathological bone formation [1,2]. In agreement with these observations, we detected both structured trabecular regions composed of parallel collagen bundles and less organized fibrillar patterns characterized by extensive branching and shorter skeleton segments.

Importantly, most previous SHG studies of bone collagen organization were performed in developmental, fracture-healing, osteoporosis, or experimental remodeling models, whereas trabecular collagen architecture within bone marrow trephine biopsies has received substantially less attention. This limitation is particularly relevant in myeloproliferative neoplasms, where fibrosis is frequently accompanied by profound alterations in bone remodeling and osteosclerotic microarchitecture [20,21,26]. Our findings suggest that SHG-based morphometric analysis may provide a quantitative framework for assessing not only fibrosis but also pathology-associated remodeling of trabecular bone collagen organization within the bone marrow microenvironment.

The highly reproducible frequency maximum of the local thickness histogram observed at approximately 3.2 μm in structured trabecular bone is remarkably consistent with the known hierarchical organization of bone collagen. Trabecular bone lamellae were previously reported to be approximately 6 μm thick [42], whereas mineralized collagen fibrils assemble into bundles of approximately 2–3 μm that form lamellar bone architecture [9]. Therefore, the SHG-derived local thickness maximum observed here may reflect a characteristic structural scale of collagen bundle organization within trabecular lamellae.

Altogether, we used four classes of metrics to characterize collagen geometry: fiber thickness, branching, branch length, and orientation dispersion, all of which demonstrated substantial differences between structured trabecular bone and the other two tissue types (unstructured trabecular bone and fibrosis). Future studies may combine multiple SHG-derived morphometric parameters into multidimensional feature spaces enabling automated classification of collagen microarchitectures.

The “unstructured” SHG pattern observed in some trabecular regions likely reflects a combination of biological and geometrical factors. On one hand, increased branching and fragmentation of the skeletonized collagen network may correspond to remodeling-associated disorganization and osteodestructive alterations of trabecular collagen architecture. On the other hand, SHG signal morphology is strongly influenced by fibril orientation relative to the imaging plane and excitation geometry. Consequently, regions containing collagen bundles oriented outside the section plane may also contribute to fragmented or poorly aligned SHG patterns. Therefore, the observed morphometric differences likely reflect both true pathological remodeling and orientation-dependent projection effects associated with complex three-dimensional collagen organization.

Dissecting the relative contributions of the biological and geometrical factors to the resulting SHG images would be a highly relevant issue to address in future studies. In that regard, measuring collagen network parameters using morphometry data from three patients and averaging over 10 ROIs per patient allows us to partially compensate for SHG dependence on fiber orientation, when comparing the microarchitecture of structured bone versus fibrosis and structured versus unstructured bone, as the resulting data represent a broad range of fiber orientation angles relative to the optical axis (Figure S3). The robustness of the proposed morphometric pipeline with respect to collagen anisotropy is supported by the very good agreement of the peak positions of fiber thickness distributions in structured bone across the three patients (Figure 6E–G), despite markedly different fiber orientation profiles in the pooled histograms (Figure S3A).

Although the present study focused on bone marrow trephine biopsies from patients with myelofibrosis, the methodological framework developed here is not limited to hematological pathology. SHG microscopy is increasingly used for the investigation of fibrosis and extracellular matrix remodeling in a broad range of tissues, including the liver, skin, lung, kidney, and tumor stroma [1,6,7,47]. Likewise, the quantitative characterization of collagen organization is becoming increasingly relevant in studies of osteoporosis, fracture healing, osteosclerosis, and pathological bone remodeling [2,9,42]. The ability of the present workflow to quantify fiber thickness distributions, branching architecture, and collagen network topology using an affordable and reproducible image analysis pipeline suggests potential applicability to a wide spectrum of fibrosis, bone-related and extracellular matrix-related pathologies. Nevertheless, the broader applicability of the proposed workflow will require validation in substantially larger patient cohorts and across additional tissue types.

The present study has several limitations. First, only three independent patients were included; therefore, the study should be regarded as a descriptive proof-of-concept investigation rather than a confirmatory clinical study. Second, ROI selection was performed subjectively based on predefined imaging criteria and consensus between two researchers, which may introduce observer-dependent variability. Third, although the proposed workflow demonstrated consistent performance across the present dataset, external validation using independent patient cohorts will be required to establish its robustness, generalizability, and potential diagnostic utility.

Furthermore, the comparison of SHG data from primary myelofibrosis samples with healthy, fibrosis-free, and disease control cohorts will be essential to establish the biological specificity, diagnostic performance, and broader applicability of the proposed workflow.

An additional limitation is the relatively limited tissue volume represented by routine diagnostic trephine biopsies, which may restrict the representativeness of the analyzed collagen microarchitecture.

The present study is also limited by the use of two-dimensional SHG images obtained from thin histological sections for skeletonization analysis. Accordingly, individual collagen branches may be truncated by tissue sectioning, and the measured branching parameters therefore reflect two-dimensional section profiles rather than complete three-dimensional collagen networks.

Finally, skeleton objects were divided into four branch-number categories (0–1, 2–10, 11–100, and >100 branches). The selection of these branch-number categories was empirical and reflected the observation that objects containing 0–1 branches constituted the vast majority of all skeletonization-derived objects. Future studies based on substantially larger datasets may allow optimization of these branch-number categories and provide a stronger biological and methodological justification for their definition. A possible extension of the present analysis would be to subdivide skeletonized objects according to additional morphological parameters besides branch number, for example, based on their total object size. Such approaches may provide complementary information on collagen network organization.

Overall, the present findings demonstrate that SHG microscopy combined with reproducible shallow machine learning-assisted morphometry enables quantitative analysis of both fibrosis and trabecular bone microarchitecture in bone marrow pathology. Beyond conventional assessment of collagen deposition, the proposed approach provides quantitative descriptors of collagen organization, branching architecture, and structural heterogeneity, thereby expanding the analytical toolbox available for studies of bone marrow remodeling. These results support the concept that collagen microarchitecture represents an informative and largely underexplored component of the bone marrow niche microenvironment.

## 4. Materials and Methods

### 4.1. Tissue Samples

Bone marrow trephine biopsy specimens were obtained from patients diagnosed with primary myelofibrosis during routine diagnostic procedures approved by the local Ethics Committee of the Russian Scientific Center of Surgery named after Academician B.V. Petrovsky (approval No. 9/27 October 2023). All samples were anonymized before image acquisition and quantitative analysis. All included patients were adults (>18 years). In every case, the diagnosis strictly fulfilled the WHO diagnostic criteria for overt primary myelofibrosis (5th edition) [48]. All bone marrow trephine biopsies were independently evaluated by four experienced hematopathologists and subsequently reviewed jointly at a multi-headed microscope. Cases were included only after reaching a consensus diagnosis. Reticulin and collagen fibrosis were assessed on separate sections stained by silver impregnation and Masson’s trichrome, respectively. In all cases, the corresponding fibrosis grades were consistent with the WHO diagnostic criteria for overt primary myelofibrosis (5th edition) [48]. Molecular confirmation constituted a mandatory inclusion criterion, and all three patients carried a driver mutation in either *JAK2* or *CALR*, thereby fulfilling the third major WHO diagnostic criterion. SHG imaging was performed on unstained sections, whereas the corresponding silver- and trichrome-stained sections from the same biopsy were used for routine histopathological diagnosis.

Trephine biopsy samples were fixed in 10% neutral buffered formalin (NBF), decalcified in EDTA, embedded in paraffin, and sectioned for microscopy. Decalcification was performed in a saturated EDTA (disodium ethylenediaminetetraacetate) solution prepared as follows. First, 150 g of disodium EDTA was dissolved in 850 mL of distilled water preheated to 50–60 °C until a slight insoluble excess remained, followed by the addition of 150 mL of 40% formaldehyde solution. The pH was adjusted to 7.0–7.5 with 40% aqueous NaOH. Decalcification was carried out at 37 °C for 4–5 days. After decalcification, the specimens were rinsed in running water for 30–60 min prior to dehydration. Sections used for second harmonic generation (SHG) imaging were cut at 2 μm thickness and mounted in Leica CV Mount medium.

Three independent primary myelofibrosis cases were included in the present exploratory morphometric study. Representative regions containing structured trabecular bone, unstructured trabecular bone and fibrosis were selected for SHG imaging and quantitative analysis.

### 4.2. Microscopy

Biopsy samples were sectioned at 2 μm thickness and mounted in the xylene-based Leica CV Mount medium (Leica Biosystems, Nussloch, Germany). Backward SHG imaging was conducted on a Leica TCS SP5MP confocal microscope (Leica Microsystems, Wetzlar, Germany) with 800 nm Mai Tai Ti:Sapphire (Spectra-Physics, Santa Clara, CA, USA) tunable laser excitation and 390–410 nm bandpass acquisition. Simultaneously, two-photon excited fluorescence (TPEF) images were acquired in the 500–700 nm emission range. Imaging was performed with an oil immersion objective (HC X PL APO CS 10×/0.40 IMM UV; Leica Microsystems, Wetzlar, Germany) at a pixel size of 200 nm. Images were acquired as single optical sections (4096 × 4096 pixels) at a scanning speed of 100 Hz with 2× frame averaging and stored in Leica (.lif) format.

### 4.3. Image Analysis

Image processing and analysis were conducted using Fiji (software version ImageJ 1.54p) [49]. Raw SHG images were used for subsequent image analysis without manual contrast enhancement or other image processing prior to segmentation. Structured trabecular bone was identified by the presence of the characteristic striped SHG pattern (Figure 1 and Figure 6), whereas unstructured trabecular bone was defined by the absence of this pattern. The bone borders were visualized using simultaneously acquired TPEF images (excitation 800 nm, emission 500–700 nm), which provided clear delineation of the trabecular boundaries. SHG-positive collagen fibers located outside the trabecular bone were classified as fibrosis. ROI selection was completed before any quantitative morphometric measurements were performed. ROIs were classified exclusively according to predefined imaging criteria (SHG morphology and TPEF visualization of trabecular boundaries), without access to any morphometric output. Therefore, the ROI classification was effectively blinded to the subsequent quantitative analysis. ROIs were selected subjectively, and inclusion in the analysis required consensus between two experienced researchers.

The segmentation quality was quantitatively evaluated using two overlap-based similarity metrics, the Dice coefficient and Intersection over Union (IoU). The images used for both the qualitative assessment of classifier performance and quantitative Dice and IoU validation were independent of those used for classifier development.

Fiji plugins were used with their default settings. For local thickness and skeleton geometry measurements 10 ROIs 80 × 80 μm size were selected for each type of SHG images—for structured bone, unstructured bone and fibrosis. Data were averaged over those 10 ROIs. A histogram bin size of two pixels was used for the local thickness analysis.

Collagen orientation was quantified using the Fiji directionality plugin (Fourier components method). The plugin estimates the dominant fiber orientation and the angular dispersion around the dominant direction by fitting the orientation histogram. LabKit-derived masks were converted from 8-bit to 16-bit format prior to the directionality analysis.

### 4.4. Statistical Analysis

Data were analyzed using estimation statistics, reporting mean paired differences with bootstrap-derived confidence intervals, rather than relying exclusively on null-hypothesis significance testing [50,51]. Standardized paired effect sizes (Cohen’s dz) were calculated to quantify within patient differences [52,53].

The present study was designed as an exploratory proof-of-concept investigation including three independent patients. Because of the limited sample size, our primary objective was the estimation of the magnitude, direction, and consistency of the observed effects across independent biological samples.

Accordingly, we adopted an estimation statistics framework, which emphasizes effect sizes and confidence intervals rather than binary significance testing [50,53]. Mean paired differences were used to quantify the magnitude of the observed changes, whereas bootstrap-derived 95% confidence intervals were calculated to estimate the uncertainty associated with these effect estimates without relying on strong distributional assumptions [50]. Standardized paired effect sizes (Cohen’s dz) were additionally reported to facilitate comparison of the effect magnitude [53].

For each paired comparison, bootstrap confidence intervals were generated from the per-patient paired differences. Specifically, the three patient-level differences were resampled with replacement 20,000 times, preserving the paired structure of the data. ROI-level measurements and individual segmented collagen objects were not resampled; these measurements were used only to obtain the corresponding per-patient mean values.

For the analyses presented in Figure 6, Figure 8, Figure 9 and Figure 10, statistical comparisons were based on averages per patient (3 patients; N = 3 independent experiments). For each patient, measurements were averaged for 10 regions of interest per tissue type (structured trabecular bone, unstructured trabecular bone, and fibrosis). For the classifier comparison shown in Figure 4 and Figure S2, measurements from three ROIs per patient were averaged. All statistical analyses in Figure 4, Figure 6, Figure 8, Figure 9, Figure 10 and Figure S2 were performed using these per-patient mean values. Thus, the patient, rather than the individual ROI or segmented collagen object, constituted the experimental unit throughout the statistical analysis. Accordingly, ROIs were treated as within-patient sampling units used to estimate tissue characteristics and were not considered independent biological replicates.

## Data Availability

The raw data supporting the conclusions of this article will be made available by the authors on request.

## Supplementary Materials

The following supporting information can be downloaded at https://www.mdpi.com/article/10.3390/ijms27177685/s1.
