# Supplementary material for: Machine Learning-Assisted SHG Morphometry Reveals Distinct Collagen Microarchitectures of Trabecular Bone and Fibrosis in Bone Marrow Biopsies
Source: Int J Mol Sci. 2026 Aug 27;27(17):7685. doi: 10.3390/ijms27177685 (PMC13566618; doi:10.3390/ijms27177685)
Supplement: Supplementary file 1 [file ijms-27-07685-s001.zip › ijms-4447700-supplementary.pdf]

Supplementary Materials

Machine learning-assisted SHG morphometry reveals distinct collagen microarchitectures of trabecular bone and fibrosis in bone marrow biopsies

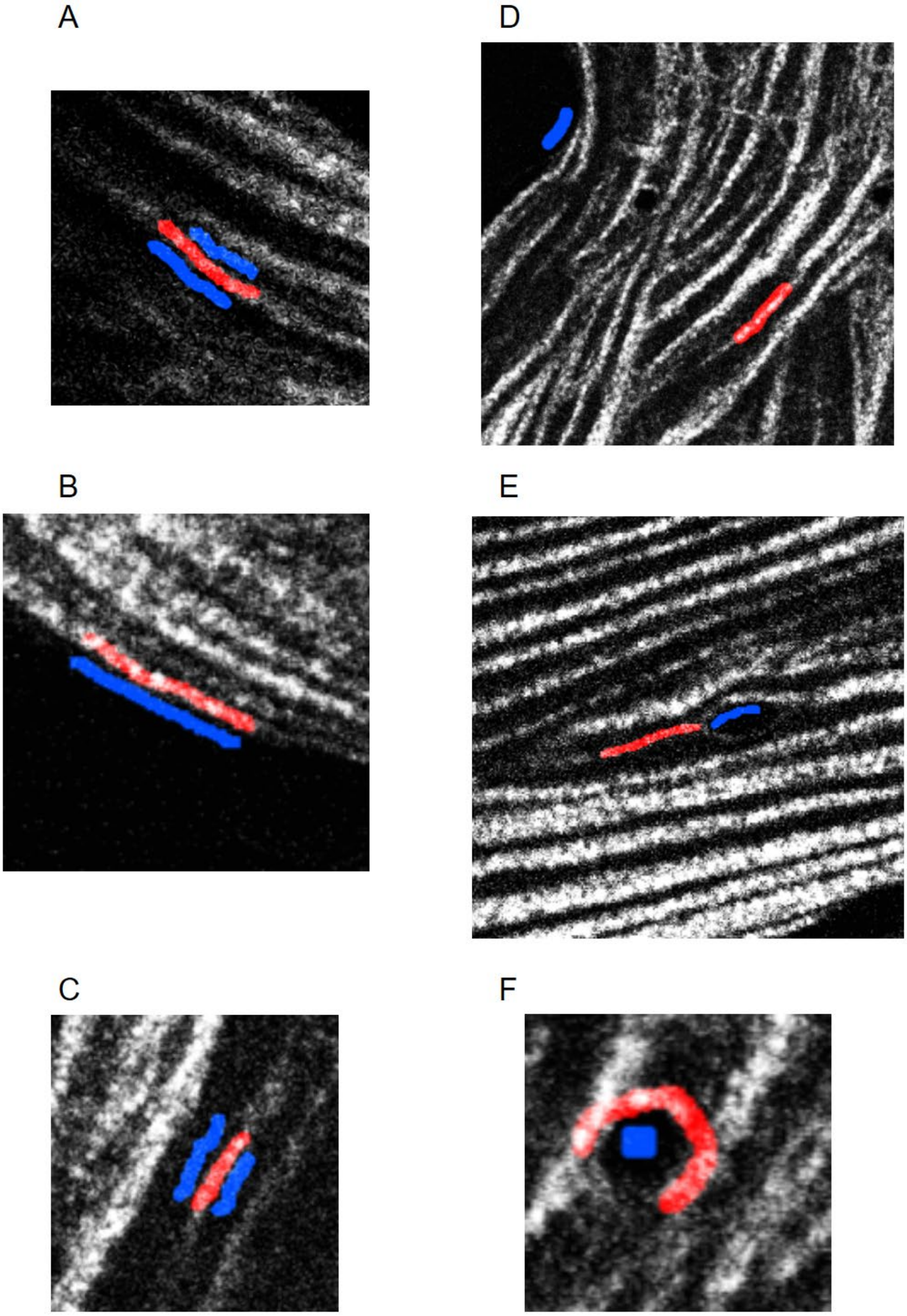
A
D
B
E
C
F

Figure S1. Additional rule-compliant (A-C) and rule-violating (D-F) classifiers created for testing outcomes of different annotation strategies.

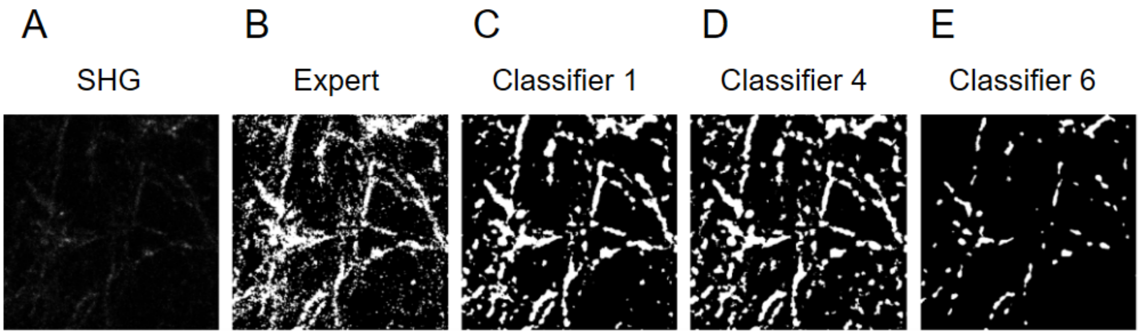

	F	G	H	I
	Clas.1 – Clas.4 Dice	Clas.1 – Clas.4 IoU	Clas.6 – Clas.4 Dice	Clas.6 – Clas.4 IoU
Mean difference (scar - nonscar)	0.07	0.06	0.3633	0.3233
Bootstrap 95% CI for mean difference	[−0.01, 0.2]	[0, 0.16]	[0.28, 0.44]	[0.3, 0.35]
Cohen's dz	0.575	0.688	4.53	12.8
Bootstrap 95% CI for dz	[−0.289, 5.88×10^{15}]	[0.577, 1.4]	[3.61, 5.44×10^{15}]	[11, 5.15×10^{15}]

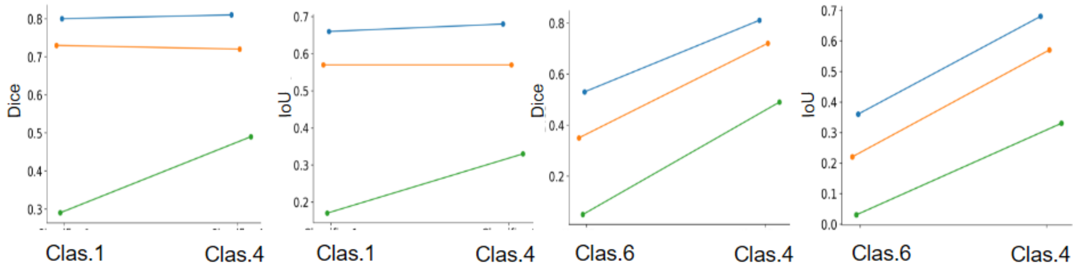

Figure S2. Comparison of segmentation performance between LabKit classifiers. A, Representative SHG image of fibrosis. B, Expert-selected threshold-based reference masks corresponding to panel A. C–E, Segmentation results obtained with classifiers 1, 4, and 6, respectively. Segmentation accuracy was evaluated using two overlap metrics, the Dice coefficient and Intersection over Union (IoU), calculated between the expert-generated reference masks and the corresponding LabKit-derived masks. The values were averaged across

three ROIs per patient, and paired bootstrap analysis was performed for classifier pairs 1 vs. 4 (F, G) and 6 vs. 4 (H, I). Data are presented as per-patient averages (N = 3 independent patients). The dataset comprised three ROIs from each patient, for a total of nine ROIs.

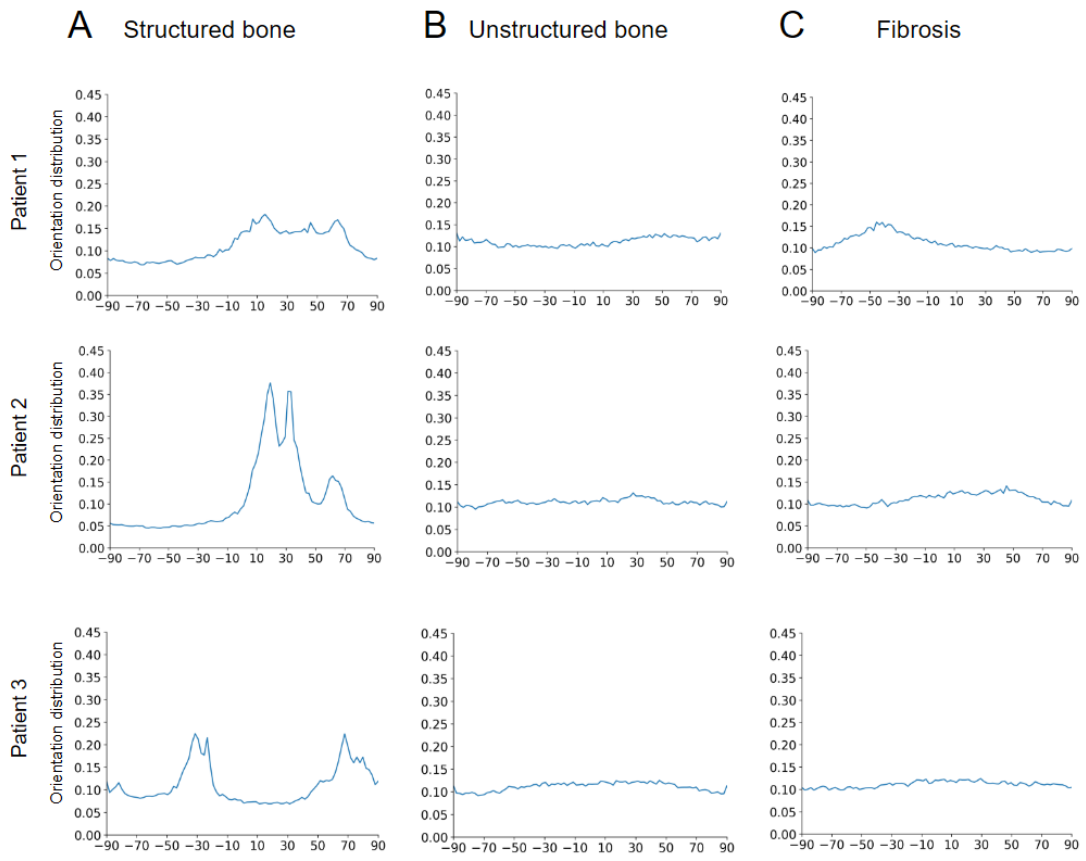

Figure S3. Pooled orientation histograms for collagen patterns of structured trabecular bone (A), unstructured trabecular bone (B), and fibrosis (C) in SHG images. Directionality distributions obtained using the Fiji Directionality plugin were summed across 10 ROIs for each tissue type and patient.
